# Effectiveness of secondary and tertiary prevention for violence against women in low and low-middle income countries: a systematic review

**DOI:** 10.1186/s12889-017-4502-6

**Published:** 2017-07-04

**Authors:** Lucy Kirk, Samantha Terry, Kamalini Lokuge, Jessica L. Watterson

**Affiliations:** 1 Research School of Population Health, The Australian National University, Building 62, Mills Road, Canberra, ACT 2601 Australia; 2 School of Public Health, University of California, Berkeley, University Hall, Berkeley, CA 94720 USA

**Keywords:** Violence against women, Intimate partner violence, Non-partner sexual violence, Secondary prevention, Tertiary prevention, Systematic review, Effectiveness

## Abstract

**Background:**

Violence against women (VAW) is a major problem worldwide, with one in three women experiencing violence in their lifetime. While interventions to prevent violence (primary prevention) are extremely important, they can take many years. This review focuses on secondary and tertiary prevention interventions that address the needs of survivors of violence and aim to prevent recurrence. This review also focuses on studies taking place in low and low-middle income countries, where rates of VAW are highest.

**Methods:**

Searches of peer-reviewed and grey literature took place from March–June 2016 through databases (Embase, CINAHL, WHO Global Index Medicus, Medline, PsychINFO, Web of Science, Cochrane Library, Applied Social Sciences Index and Abstracts and Sociological Abstracts) and by consulting experts in the field. Only primary research was eligible for inclusion and studies had to focus on secondary or tertiary prevention for survivors of VAW in low or low-middle income countries. All study designs were eligible, as long as the study examined client-related outcome measures (e.g., incidence of violence, health outcomes or client satisfaction). Data were extracted and quality of the studies was assessed using the Effective Public Health Practice Project Quality Assessment Tool for Quantitative Studies and a qualitative quality assessment tool developed by Mays and Pope. Due to the low number of results and heterogeneity of the study populations and outcomes, a narrative synthesis was conducted and evidence was summarized.

**Results:**

One thousand two hundred fifteen studies were identified through the search strategy and 22 of these met the eligibility criteria. Overall, the evidence for interventions is weak and study limitations prevent definitive conclusions on what works. There is some evidence that interventions targeting alcohol use, both among perpetrators and survivors, may be effective at reducing VAW through secondary prevention, and that psychotherapy might be effective for survivors of non-partner sexual violence through tertiary prevention. Finally, some evidence exists for crisis centres increasing survivors’ access to services (through both secondary and tertiary prevention), however, assessment of their impact on future VAW are needed.

**Conclusions:**

Though some interventions for survivors of VAW have shown evidence of effectiveness, further research is needed, especially high-quality studies with quantitative outcome data.

**Electronic supplementary material:**

The online version of this article (doi:10.1186/s12889-017-4502-6) contains supplementary material, which is available to authorized users.

## Background

Violence against women (VAW) is a major issue worldwide, with an estimated 35% of women, or roughly 1 in 3, experiencing either physical and/or sexual intimate partner violence or non-partner sexual violence in their lifetime [1]. The majority of violence against women is intimate partner violence (IPV), which includes physical or sexual violence occurring within an intimate relationship, such as marriage or dating [2]. The second major type of VAW is non-partner sexual violence, experienced by at least 7% of women in their lifetime [3]. Non-partner sexual violence includes rape, sexual assault, and any other violence of a sexual nature perpetrated by someone who is not the victim’s intimate partner [2].

Women experiencing violence face increased preventable morbidity and mortality, making VAW a pressing public health concern. Both forms of VAW can lead to serious short and long-term harm for survivors [1, 4]. Psychological effects include post-traumatic stress disorder, anxiety, depression and increased rates of suicide [1, 5–7]. Physical and reproductive effects include increased risk of transmission sexually transmitted infections, including Human Immunodeficiency Virus (HIV), unwanted pregnancy, poor maternal outcomes, pain and injuries, among many others [1, 8–11]. All women who experience violence are at risk of repeated occurrence of this violence, particularly so for those experiencing IPV.

**Fig. 1:**
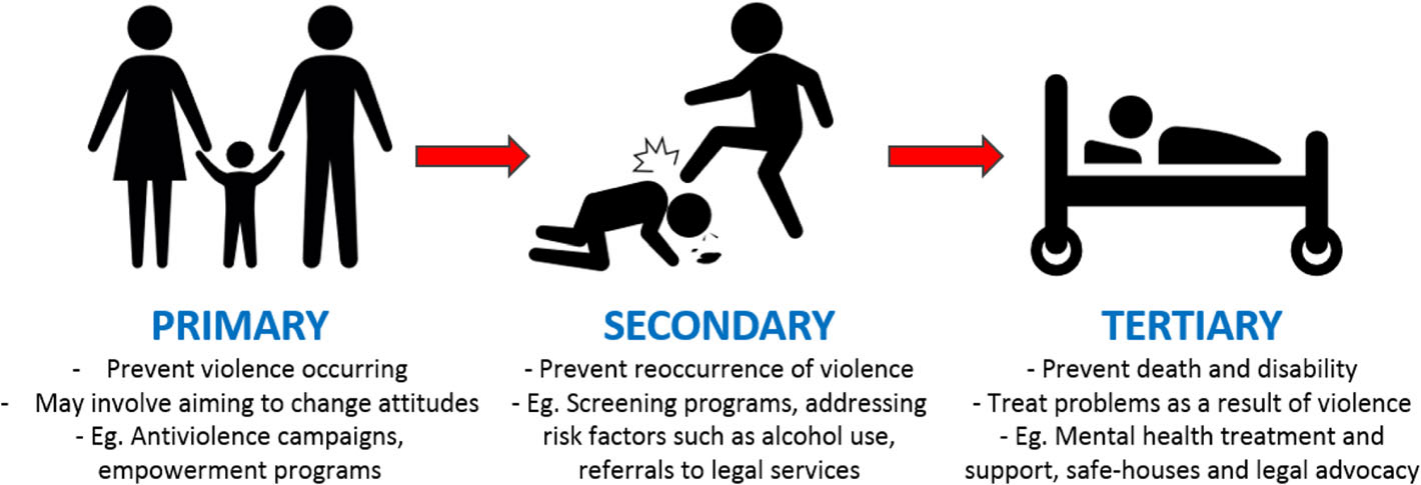
Prevention of violence against women at primary, secondary and tertiary levels

Public health interventions, such as those aimed to address VAW, can be divided into three types: primary, secondary or tertiary prevention (Fig. 1) [12, 13]. Primary prevention aims to prevent the disease or health event occurring, while secondary prevention aims to detect the issue early and prevent progression or reoccurrence of the event. Finally, tertiary prevention aims to prevent death and disability associated with the disease or health event [12, 13]. Though more recent prevention models have been developed, such as the 1994 Institute of Medicine prevention classification system, we chose to use this model, as it is well-suited to a review that addresses interventions and services for the subset of women already experiencing violence [14]. Primary prevention is undoubtedly critical for long-term reduction of VAW. However, the process of changing deep-rooted societal beliefs is never fast, and the current need for secondary and tertiary interventions to address VAW is acute.

A WHO multi-country study found that in general, VAW is more prevalent in low and middle income countries than in high income countries [2]. Lifetime prevalence of VAW is estimated to range from 32.7% in high-income countries to 45.6% in the African region [2]. Low and middle income countries are also the settings which are likely to experience the most challenges in resourcing services for survivors. Although further research is needed into interventions that reduce risk for women experiencing violence even in high-income countries, some evidence on what is effective does exist [15]. It is essential to determine if these interventions are transferrable to lower-resourced settings. Therefore, there is a critical need to identify interventions that are effective in addressing the physical and mental health needs and preventing further violence in low-income settings. The aim of this systematic review was to compile and evaluate existing evidence for secondary and tertiary interventions to address VAW in low and low-middle income countries.

## Methods

### Inclusion and exclusion criteria

Studies eligible for inclusion were those that focused on secondary or tertiary prevention for female survivors of IPV or non-partner sexual violence, and were conducted in a country defined as low or low-middle income by the World Bank [16]. Included studies also had to be an evaluation or analysis of intervention effectiveness that examined some outcome measures (e.g., incidence of violence, health outcomes or experiences of survivors, client satisfaction or quality of care), not only process measures. Due to the fact that randomised trials are often not available for system or cross-sectoral interventions, such as those for violence against women, any type of study design was eligible for inclusion [17]. Studies were excluded if the intervention was targeted at women who had not previously experienced violence or men who were not perpetrators of violence (i.e., primary prevention). Only primary research articles were included, but studies from both peer-reviewed literature and grey literature were eligible for inclusion. The inclusion criterion of primary research excluded articles such as commentaries and letters to the editor. The bibliographies of identified reviews were screened for relevant articles. No restrictions were applied to the dates of studies, to the age of participants or to the language of the article, however, searches were only performed using English search terms, which could have resulted in a de facto language restriction.

### Searching and screening strategy

Searches were conducted separately for the two major areas of VAW: intimate partner violence (IPV) and non-partner sexual violence. The search strategy involved systematic searches of 8 medical and social science databases between March and June 2016. The following databases were searched for literature on both IPV and non-partner sexual violence: Embase, CINAHL, WHO Global Index Medicus, Medline, PsychINFO, Web of Science, The Cochrane Library, Applied Social Sciences Index and Abstracts (ASSIA) and Sociological Abstracts. A search on IPV only was also performed in one additional database: International Bibliography of the Social Sciences (IBSS). A search for grey literature on non-partner sexual violence was also performed using the databases Opengrey and Greylit, as well as Google and Google Scholar (top 200 results only).

The search terms and their combinations (Table 1) were adapted to the search features of each database. Actual search terms and minor variations are outlined in Additional file 1. Experts in the field of VAW were also contacted in order to identify relevant studies.

**Table 1:** Search terms and their combinations

Population	Health issue	Intervention	Evaluation	Location
female^a^ women^a^ girl^a^ wife^b^ partner^b^ child^c^	sexual abuse^a^ sexual violence^a^ intimate partner violence^b^ abuse^b^ domestic violence^b^ sexual assault^c^ sexual coercion^c^ rape^c^ sexual harassment^c^	treatment^a^ secondary prevention^a^ tertiary prevention^a^ response^c^ best practice^c^	efficacy^a^ effectiveness^a^ outcome^a^ measure^c^ evaluation^c^ what works^c^ impact^c^	All countries listed as low or low-middle income by the World Bank^a^

^a^used in all searches

^b^used in searches for IPV only

^c^used in searches for non-partner sexual violence only

Following compilation of all articles resulting from this search strategy, 245 abstracts were independently co-screened by LK and ST, with inter-rater reliability of 97.1%, indicating clarity of the inclusion criteria. The seven discrepant ratings were resolved by discussion to reach a consensus, then the remaining articles were screened independently.

### Data extraction, analysis and quality assessment

The quality of included quantitative studies was assessed using the Effective Public Health Practice Project (EPHPP) Quality Assessment Tool for Quantitative Studies (QATQS) [18]. Previous evaluation of this tool has shown it to be valid and reliable [19]. The EPHPP-QATQS was used to assess the risk of bias within individual studies by considering eight components of study methodology in order to generate an overall quality rating of weak, moderate or strong. The components of study methodology considered were selection bias, study design, presence of confounders, blinding of participants and outcome assessors, validity and reliability of data collection methods and study dropouts and withdrawals. Quality assessment was performed by two reviewers: for quantitative studies on IPV, LK and KL assessed quality and for quantitative studies on non-partner sexual violence, ST and KL assessed quality. Inter-rater reliability was found to be 91% and the one discrepant rating was resolved through discussion.

Data were extracted from all quantitative studies on IPV by one reviewer (LK), and all quantitative studies on non-partner sexual violence by another reviewer (ST). The data extraction form was based on recommended data for inclusion from the Cochrane Handbook [20].

The quality of included qualitative studies was assessed using a tool created by Mays and Pope; the specific criteria are outlined in Table 2 [21], modelled after a table used in a qualitative systematic review by Robinson & Spilsbury [22]. Despite ongoing debates about whether the quality of qualitative research can or should be assessed, the Cochrane Collaboration Qualitative Methods Group recommends that critical appraisal of qualitative studies should be included an intervention review [23]. Further, no qualitative studies were excluded for methodological reasons, and all studies exhibited some strengths that made the results worth including. One reviewer (JLW) extracted information from qualitative studies and assessed quality.

**Table 2:** Methodological quality of included qualitative studies

Key: 0 = low clarity and quality as assessed by the reviewer 1 = reasonable clarity and quality as assessed by the reviewer 2 = reflects a finding of high clarity and quality as assessed by the reviewer NC = not clear or not available from the paper	Bernath 2013 [24]	Bhate-Deosthali 2012 [44]	Doucet 2012 [43]	GHD Pty Ltd. 2015 [25]	Human Rights Watch 2015 [26]	Keesbury 2012 [27]	Kohli 2013 [46]	Manneschmidt 2009 [42]	Morel-Seytoux 2010 [28]	PHD Group 2012 [29]	Wessel 1997 [45]
1) Worth or relevance											
1.1) Was this piece of work worth doing at all?	2	2	2	2	2	2	2	2	2	2	2
1.2) Has it contributed usefully to knowledge?	2	2	2	2	2	2	2	2	2	2	2
2) Clarity of research question											
2.1) If not at the outset of the study, by the end of the research process, was the research question clear?	2	1	2	2	1	2	2	2	2	2	1
3) Appropriateness of the design of the question											
3.1) Was an appropriate method used?	1	NC	1	1	1	2	2	1	1	1	1
4) Context											
4.1) Is the context or setting adequately described so that the reader could relate the findings to other settings?	2	2	2	1	2	2	2	2	2	2	2
5) Sampling											
5.1) Did the sample include the full range of possible causes or settings?	0	NC	0	NC	NC	1	1	NC	NC	NC	1
5.2) If appropriate, were efforts made to obtain data that might contradict or modify the analysis extending or modifying the sample?	1	NC	0	NC	NC	NC	NC	NC	NC	NC	NC
6) Data collection and analysis											
6.1) Were the data collection and analysis procedures systematic?	1	NC	2	NC	NC	1	2	1	0	0	1
6.2) Was an ‘audit trail’ provided?	1	0	2	1	0	1	1	1	1	0	0
6.3) How well did the analysis succeed in incorporating all the observations?	NC	NC	NC	NC	NC	NC	NC	2	2	NC	NC
6.4) Did the analysis develop concepts and categories capable of explaining key processes?	2	1	2	2	2	2	2	2	2	NC	2
6.5) Was it possible to follow iteration between data and theory?	2	0	2	1	NC	2	2	2	1	0	2
6.6) Did the researcher search for disconfirming cases?	0	NC	0	NC	NC	NC	NC	NC	NC	NC	NC
7) Reflexivity of the account											
7.1) Did the researcher assess the likely impact of the methods used on the data obtained?	0	0	2	0	0	0	0	0	2	0	0
7.2) Were sufficient data included in the reports to provide sufficient evidence for readers to assess whether analytical criteria were met?	0	1	2	2	2	2	2	1	1	0	2

Due to the heterogeneity of the studies selected, a meta-analysis of data could not be conducted. Data and information extracted was synthesised in a narrative review.

## Results

**Fig. 2:**
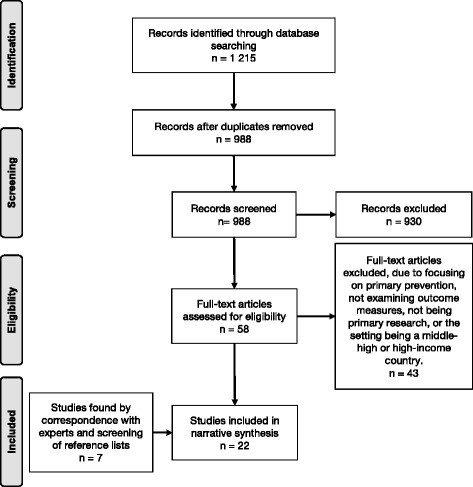
PRISMA flowchart of screening and selection

The search generated a total of 1215 records for further screening, with 988 remaining after removal of duplicates (Fig. 2). Following independent co-screening of 245 abstracts with high inter-rater reliability, the remaining 743 abstracts were screened independently. A total of 22 studies were included in this review. Half (*n* = 11) of these used mostly quantitative methods and had a total of 2185 participants. The other half of the included studies (*n* = 11) used mostly qualitative methods (see Tables 3, 4 and 5). Given that this review was focused on the effectiveness of interventions, we have focused on outcome data in the results and narrative synthesis (i.e., not process measures). Six (27%) of the included studies were from the grey literature [24–29] and the other 16 studies (73%) were from peer-reviewed journals. The majority of studies took place in Africa (*n* = 13, 59%), followed by Asia and the Pacific (*n* = 8, 36%), and only one study (5%) took place in Latin America (see Tables 3, 4 and 5).

**Table 3:** Overview of quantitative studies focusing mostly on intimate partner violence (IPV)

Study	Country and setting	Study design and sample	Intervention	Outcomes	Global quality rating
Saggurti et al. 2014 [34]	Mumbai, IndiaLow-income community (slum)	Cluster-randomised controlled trial. Married women reporting IPV or that their husband engages in heavy drinking were enrolled in the intervention or control group based on their geographic cluster. Control group *n* = 102 Intervention group *n* = 118	The *Reducing HIV among Non-Infected Wives (RHANI)* program included four individual sessions and two group sessions over 6–9 weeks. Sessions focused on problem solving and marital communication, as well as building social cohesion (group sessions). The control group received referrals to local services, and both groups viewed street plays about marital violence and alcohol use in their villages.	Intention-to-treat analysis of survey measures at baseline and follow-up. A reduction in self-reported marital conflict in the last 3 months was seen for the intervention group, but results were not statistically significant at a *p*-value of 0.05 (RR = 0.4; 90% CI 0.1–0.9; *p* = 0.064). Similarly no statistically significant effect was seen on marital IPV in the last 3 months (RR = 0.7; 90% CI 0.2–1.8; *p* = 0.548) or sexual coercion in the last 3 months (RR = 0.2; 90% CI 0.05–0.9; *p* = 0.082).	WEAK
Satyanarayana et al. 2016 [39]	Bangalore, IndiaInpatient hospital psychiatric services	Randomised controlled trial. Male patients admitted to psychiatric services with Alcohol Dependency Syndrome (ADS), who were married with children, and admitted to perpetration of IPV were randomized: Control group *n* = 88Intervention group *n* = 89	The *Integrated Cognitive Behavioural Intervention (ICBI)* consisted of eight sessions discussing links between alcohol and IPV, consequences and prevention of IPV, as well as teaching of cognitive behavioural techniques such as anger management. The control group received treatment as usual, consisting of pharmacotherapy and psychoeducation regarding treatment ADS.	Survey measures (from both husband and wife) at baseline, 1 month follow-up and 3 months follow-up. Though no statistically significant reduction in alcohol consumption was found among the intervention group relative to the control group at 3 months post-intervention (*p* = 0.44), significant reductions in the wives’ reports of violence (Effect size = 0.24; *p* = 0.005) and symptoms of depression (Effect size = 0.17; *p* = 0.04), anxiety (Effect size = 0.15; *p* = 0.006) and stress (Effect size = 0.07; *p* = 0.01) were seen, relative to the control group.	MODERATE

**Table 4:** Overview of quantitative studies focusing mostly on non-partner sexual violence

Study	Country and setting	Study design and sample	Intervention	Outcomes	Global quality rating
Allon 2015 [30]	Democratic Republic of the Congo Treatment sites in the towns of Kakwende and Kasika	Controlled clinical trial. Female Congolese victims of sexual violence were enrolled in one of two therapies, based on provider’s opinion of most appropriate treatment. Individual therapy *n* = 8 Group therapy *n* = 28	The individual therapy consisted of 2 sessions of eye movement desensitisation and reprocessing (EMDR) therapy. The group therapy consisted of 2 sessions of modified EMDR-Integrative Group Treatment Protocol (IGTP). Both therapies were delivered by a visiting Israeli doctor.	Subjective intensity of distress measured pre-and immediately post-treatment using SUD score. Mean SUD score decreased from 9.0 (±1.3) to 4.8 (±2.9) immediately post-treatment for EMDR-IGTP group (*p* < 0.0001), and from 9.3 (±0.9) to 1.9 (±2.2) for individual therapy group. NSD between pre-intervention SUD scores between individual and EMDR-IGTP group, but significant difference post-intervention (*p* < 0.01), with individual therapy more effective in lowering SUD scores. IES scores decreased from mean of 52 before group therapy to 33 afterwards (*n* = 6), *p* < 0.03.	MODERATE
Bass et al. 2013 [31]	Democratic Republic of the Congo NGO offices in 14 villages in South Kivu and 2 villages in North Kivu provinces	Cohort study (two groups). 6 village clusters were randomized to receive one of two therapies and female survivors of sexual assault with clinically significant psychological problems were enrolled. Cognitive processing therapy group *n* = 157 Individual support comparison group *n* = 248	Cognitive processing therapy consisted of 1 individual 1-h session and 11 group sessions with 6–8 women each. Women in the individual support comparison group were invited to access individual psychosocial and case-management support as desired.	Depression and anxiety symptoms assessed by a questionnaire administered pre-treatment, immediately post and 6-months post treatment. Group therapy: probable PTSD reduced from 60% prevalence pre-treatment to 8% post-treatment and 9% 6 months later (*p* < 0.001). These measures were 83% to 54% to 42% for individual support (*p* < 0.001). Probable depression or anxiety reduced from 71% to 10%, then 9% at 6 months for group therapy (*p* < 0.001), and 83% to 54% to 42% for individual support (*p* < 0.001). Symptom improvements significantly greater for group therapy compared to individual support.	STRONG
Deb, Mukherjee, and Mathews 2011 [37]	Kolkata, India Schools and shelters	Cross-sectional study. Sexually-abused girls, aged 13–18, were purposively selected from 4 randomly selected shelters for the intervention group. A comparison group of non-sexually abused girls of the same ages were randomly selected from 4 nearby schools. Comparison group *n* = 120 Intervention group *n* = 120	Sexually-abused girls received a minimum of weekly individual and group counselling for at least 2 months.	Among sexually abused girls, 58.3% found counselling to be beneficial. A statistically significant difference in aggression was seen between sexually-abused girls who found counselling to be beneficial and sexually-abused girls who did not find counselling beneficial. Difference between mean aggression scores 14.00 95% CI 8.12, 21.68 *p* = 0.00	WEAK
Hall et al. 2014 [32]	As in Bass et al. 2013	As in Bass et al. 2013	As in Bass et al. 2013	Social capital measured using a questionnaire administered pre and post intervention. Group therapy associated with increased group membership and participation (*p* < 0.05) at 6 month follow up. Emotional support seeking increased from pre to post- intervention (*p* < 0.05) but was not maintained at 6 months. NSD between group therapy and individual support for contact with non-kin social networks, instrumental support network size or financial network size.	STRONG
Hogwood et al. 2014 [35]	Rwanda	Cohort study (one group). Rwandan women caring for their children born from rape were purposively selected to receive the intervention, mainly based on their geographic location and receipt of previous support from a local NGO. *n* = 40	Twelve fortnightly counselling groups of 10 members, led by female graduate-level trained Rwandan counsellors. The aims of the counselling groups were to encourage within-group social support, address emotional pain, assist in disclosure of rape to children, improve parenting skills and relationships.	Questionnaire administered pre-intervention, at halfway, post-intervention and 3 months post. Participants rated groups as helpful (mean 7/10 at mid-point, 9/10 at end). Life satisfaction increased between time one, two and three (*p* < 0.0005) but decreased at follow up (*p* < 0.005). 65% increase in social support over course of intervention (*p* < 0.0005). Acceptance of being a parent to the child, and reporting of “very good” relationship with child increased 47% and 33% respectively (*p* < 0.0005).	WEAK
Hustache et al. 2009 [36]	Republic of the Congo MSF clinic in Brazzaville, during a period of conflict (2002–2003)	Cohort study (one group). Women over age 15, raped by unknown military personnel were enrolled. *n* = 64	Individual psychological counselling offered as part of post-rape care, specifically addressing - social and familial concerns - coping strategies - acceptance, future plans	Global functioning: Medium-extreme impairment in global functioning in 89.3% participants pre-intervention and 28.6% post-intervention, *p* = 0.04. Effect maintained 1–2 years post-treatment. However 31.3% participants report familial detachment, 3.1% met PTSD diagnostic criteria, 40.6% report re-experiencing symptoms 1–2 years after intervention.	WEAK
Lekskes, van Hooren, and de Beus 2007 [33]	Liberia Rural villages	Controlled clinical trial. Liberian women who had experienced sexual violence during conflict were enrolled in one of two intervention groups, or the waiting list control group. Waiting list control group *n* = 21 Trauma counselling group *n* = 58 Support and skills training group *n* = 54	The trauma counselling group received a 3-month program, consisting of 8 individual sessions and group counselling. The support and skills training group received skill training to support income generation, and discussed gender issues and sexual abuse. The waiting list control group were pre-selected for either intervention.	Decrease in PTSD score (from 2.6 to 2.0) from pre-intervention to immediately post-intervention for the counselling group. Slight increase in PTSD score (1.5 to 1.7) for WHDP group. Increase in PTSD score for control group (2.0 to 2.5). (Significance not described) Reduction in PTSD scores for women in both interventions if initial PTSD score was high (statistics not described).	WEAK
O’Callaghan et al. 2013 [38]	Democratic Republic of the Congo Secondary school in the town of Beni, North Kivu province	Randomised controlled trial. 12–17 year old female Congolese victims or witnesses of sexual abuse were randomized to the intervention or control groups: Control group *n* = 28 Intervention group *n* = 24	Group based, culturally modified Cognitive Behaviour Therapy (CBT) was delivered to the intervention group for 2 h, 3 days/week for five weeks. The control group was waitlisted for the intervention.	PTSD, depression and anxiety symptoms assessed using validated measures, pre-intervention post-intervention and 3-months post. Greater improvements across all measures in intervention group compared to control (*p* < 0.001 for PTSD, Depression +Anxiety and conduct, *p* < 0–.024 for prosocial behaviour). Highly significant improvement in symptoms of PTSD with large effect size (*P* < 0.001, d = 2.04) anxiety and depression (*p* < 0.001, d = 2.45) conduct problems (*p* < 0.001, d = 0.95) and pro-social behaviour (*p* < 0.001, d = −1.57) between pre-intervention and 3 month follow up.	STRONG
Parcesepe et al. 2016 [40]	Mombasa, Kenya HIV prevention drop-in centres	Randomised controlled trial. Women over 18 who engaged in transactional sex in the past 6 months, were moderate risk drinkers and visited a HIV prevention drop-in centre were randomised to the intervention or control groups: Control group *n* = 408 Intervention group *n* = 410	WHO’s Brief Intervention for Hazardous and Harmful Drinking, adapted for the context of alcohol use and sex work, was delivered to the intervention group through 6 monthly individual sessions with trained nurse counsellors. The control group received 6 monthly individual sessions with trained nurse counsellors, focused on non-alcohol related nutrition information.	Questionnaire administered pre-intervention, immediately post-intervention and 6 months post. Compared to the control group, the intervention group experienced significant decreases in physical violence from paying sexual partners in the last 30 days, 6 months post-intervention (OR = 0.45, 95% CI 0.23–0.85, *p* = 0.01). The intervention group also experienced significant reductions in physical violence from non-paying partners in the last 30 days at 6 months post-intervention, compared to the control group (OR = 0.57, 95% CI 0.38–0.92, *p* = 0.02).	STRONG

**Table 5:** Overview of qualitative studies

Study	Country and setting	Methods	Intervention	Findings
Bernath and Gahongayire 2013 [24]	Kigali, Rwanda One stop centre in Kacyiru Police Hospital	Mixed-methods evaluation included: - desk review of existing policies, laws, etc. - collection of existing statistical data - interviews with staff and stakeholders - interviews with clients	ISANGE One Stop Centre (IOSC) is a programme designed to provide psychosocial, medical, police and legal services to survivors of abuse. It is housed inside a public hospital and offers free 24/7 service.	The quality and availability of medical and forensic services are very high and strong links are present with police. However, weaknesses include: - Difficulty following-up with survivors - Inconsistent legal aid for survivors - Requirement for survivors to report abuse to police to obtain services - Limited data collection and monitoring - Staff training is not systematically planned or monitored
Bhate-Deosthali, Sundari Ravindran, and Vindhya 2012 [44]	Mumbai, India Crisis centres at two public hospitals	Mixed-methods evaluation included: - Review of project documents - Interviews with program and hospital staff - Analysis of case records	The Dilaasa Crisis Centres offer counselling (informed by a feminist perspective) to women who have experienced violence and are referred from the hospital or other health facility. They also provide referrals to partner organizations that provide legal assistance and temporary or permanent shelter.	The centres’ locations in public hospitals make it possible to reach women from low-income or marginalized groups. Many survivors cited obtaining emotional support from the counselling and some reported improved psychological health. Survivors also stated that the centres helped them to register their complaints with the police. However, the centres faced an ongoing challenge while trying to change the attitudes of health professionals to recognize that domestic violence is an issue they should be concerned about, through ongoing training.
Doucet and Denov 2012 [43]	Sierra Leone Rural area in south	6 war-affected women and 4 social workers were purposively selected for open-ended interviews.	Social workers provided psychosocial support to women following the war. The social workers focused on giving advice, as well as principles of solidarity and spirituality in their psychosocial support, rather than clinical diagnostics and psychology.	War-affected women cited the social workers’ advice and support as playing an important role in their recovery. No women mentioned foreign professionals as making an impact on recovery, suggesting that local social work practices are valuable, despite being very different from those used in the Global North.
Human Rights Watch 2015 [26]	Papua New Guinea	46 interviews were conducted: 27 with survivors of family violence and the remainder with local officials, activists, NGO workers, and other stakeholders.	Interventions include: - Family and Sexual Violence Units (FSVU) in 17 police stations to make police more accessible to victims of gender-based violence - Family Support Centres in 15 hospitals to assist patients seeking care as a result of family violence - Hotline that provides counselling, information, guidance, and referrals for care at local services - Referral Pathway established by government officials in urban areas, designed to ensure that if a survivor accesses one service, they are linked to all other relevant ones	Many of these interventions are relatively new and are said to be effective at increasing support for survivors and access to services. However, barriers remain including: - Lack of awareness about available services - Limited access to services in rural areas - Shortage of safe houses - Service providers not putting the survivor’s best interests first (e.g., encouraging reconciliation) - Lack of psychosocial counselling and case management services - Weak law enforcement response. One expert stated that only 7 of the FSVU are functional. - Limited court capacity and legal assistance for survivors - Limited economic opportunities for survivors who leave their partners
Keesbury et al. 2012 [27]	Kenya and Zambia One-stop centres (OSC)	A comparative case study was conducted with purposively selected OSCs: 2 in Kenya and 3 in Zambia, representing a range of approaches to the OSC model. Mixed-methods were used, including: - Facility inventory - Record reviews - Key informant interviews	One-stop centres (OSCs) provide integrated, multidisciplinary services for survivors of sexual and gender-based violence in a single physical location. Three major types of OSCs exist: - Health facility-based and hospital-owned OSC, where OSC functions are integrated into routine health centre activities - Health facility-based and NGO-owned OSC, where the NGO provides “wrap around” services at the health facility - Stand alone NGO-owned OSC, which provides legal and psychosocial support onsite but refers elsewhere for medical care	The health facility-based and hospital-owned OSC model was found to be better set up to achieve a broader range of legal and health outcomes for survivors, and survivors felt that they were meeting their health needs. Challenges still remain with the OSC model: - Poor integration of OSCs with the legal system - Strengthening of the range of services provided by OSCs and/or linkages to outside services is needed
Kohli et al. 2013 [46]	Democratic Republic of Congo Two villages in Walungu Territory	In-depth interviews were conducted with 27 participants, including 13 survivors of sexual violence who were rejected by their families, 3 spouses of survivors, 1 community member, 5 mediators and 5 service providers	Family mediation is a process of resolving family conflict between family members who have rejected a survivor of sexual violence and the survivor.	Reintegrated survivors reported better relationships, improved opportunities for their children, and fewer mental health problems. However, challenges still exist, especially in cases where the survivor has a child from her rapist, or the survivor’s partner has remarried. Additional services such as economic support (e.g., children’s tuition, livelihood training, etc.) were cited as potential ways to improve reintegration.
GHD Pty Ltd. 2015 [25]	Papua New Guinea	In-depth interviews and/or focus group were conducted with staff, survivors, police officers, referral partners and other stakeholders. Limited quantitative data was also aggregated and analysed.	The Family and Sexual Violence Units (FSVU) are police units that are tasked with responding to the needs of family and sexual violence survivors. They are present in 15 police stations and also provide referrals to other services for these survivors.	The FSVUs have begun to change police response to FSV in PNG, however limitations still remain, including: - Persistent culture of male dominance in police force - Further training needed on how to provide supportive, non-judgemental services for survivors - Improvements to procedures are needed, including timeliness of response, communicating their roles and processes to survivors and making arrest and prosecution protocols consistent across police stations
Manneschmidt and Griese 2009 [42]	Afghanistan	109 women who were survivors of war-related violence participated in group evaluations (maximum of 13 participants per group), giving feedback on the intervention	Basic Counselling Training (BCT) uses a group counselling process to provide women with psycho-education, relief from distressing symptoms, new social skills (e.g., problem-solving skills) and new support networks.	Over half of participants mentioned that their social life had improved after the intervention, including their interactions with family members and their stress levels. Many participants also cited being happier or an improvement to their health.
Morel-Seytoux et al. 2010 [28]	Zambia	Mixed-methods evaluation included: - Desk review of 36 USAID and CDC monitoring and reporting documents - Key informant interviews with 240 beneficiaries, stakeholders, and ministry officials - 24 site visits/observations	Coordinated Response Centres (CRCs) in 7 districts provide care and support for survivors to meet their medical, psychological and legal needs.	The coordinated approach is an effective model and provides survivors with more comprehensive services. Coupling of these direct services with public awareness campaigns has improved the public’s knowledge of GBV and “broken the silence”
The Population Health and Development (PHD) Group Pvt. Ltd. 2012 [29]	Nepal Mobile camps in conflict-affected areas	Mixed-methods evaluation included: - In-depth interviews and focus groups - Analysis of secondary data and reviews - Field visits	Mobile reproductive health camps were conducted for six days (plus four days follow-up) in 14 of the most conflict-affected areas of the country. Survivors of SGBV presenting to the camps were offered psychosocial counselling, legal and medical services, as well as shelter.	The use of reproductive health camps to identify survivors was successful at minimizing stigma for survivors of sexual violence. 86% of survey respondents said the camp services were good and reasons cited included free drugs and services, no wait time, good provider behaviour and good counselling. The camps successfully reached marginalised populations with over 66% of clients coming from disadvantaged communities. However, challenges arose, including: - Difficulty filing complaints with police because of politics, fear of family discord, etc. - Challenges tracking survivors because the camps were conducted 5 years after the end of conflict
Wessel and Campbell 1997 [45]	Managua, Nicaragua Women’s centres in 3 poor neighbourhoods	Interviews with 21 survivors of domestic violence and 15 key informants involved in women’s centres or related projects.	The Inter-Collective is a group of three women’s centres, or *casas de la mujer,* running programs for survivors of domestic violence, including self defence classes, self-help groups, legal information workshops, health care for survivors, as well as professional services from lawyers, psychologists and nurses.	As a result of the intervention, survivors cited having new perceptions of women’s roles, increased emotional support and knowledge about their health and legal rights, decreased violence by their partners, and increased involvement in programs to help other survivors.

### Target populations

Among the 11 quantitative studies included, only two studies (18%) focused on intimate partner violence (see Table 3) and the remaining 9 (82%) focused on non-partner sexual violence (see Table 4). The 11 qualitative studies (see Table 5) could not be easily sorted into these categories because many of them did have specific inclusion criteria for their participants, based on their experience of violence. The qualitative studies mostly evaluated programs or interventions that were open to survivors of many forms of VAW.

In addition to the type of violence that these studies examined, there was also some variation in the specific target population that the intervention was delivered to. Among the targeted quantitative studies, seven studies (64%) focused on adult female survivors of VAW (and some teens over 15 were included as “adults”) [30–36]. Two studies (18%) delivered their intervention specifically to teenage girls [37, 38], while one study targeted male perpetrators of intimate partner violence [39], and a final study focused on female sex workers [40].

### Interventions

The most commonly studied intervention was psychotherapy or counselling, with 13 studies (59%) examining various forms of this intervention. Two of these studies studied the effects of Cognitive Behavioural Therapy (CBT) [38, 39], which is used for a variety of emotional, behavioural and psychiatric issues and helps patients to identify negative thoughts and behaviours and to replace them with healthier ones [41]. Another two studied Cognitive Processing Therapy (CPT) [31, 32], which is commonly used to treat depression, anxiety and post-traumatic stress disorder (PTSD) in survivors of sexual violence, and it targets unhelpful beliefs and avoidance behaviour [32]. One study evaluated the effects of Eye Movement Desensitisation and Reprocessing (EMDR), which was developed to treat people suffering from PTSD by having the client recall the traumatic event while the therapist or client apply stimulation to alternating sides of the body or eyes [30]. The remaining 8 studies examined other psychosocial or counselling interventions that did not fit specific categories of therapies [33–37, 40, 42, 43].

The second most commonly studied intervention was crisis centres, which were the focus of 6 studies (27%), all using mostly qualitative data to study outcomes [24, 27–29, 44, 45]. These crisis centres provide survivors of VAW with comprehensive, multidisciplinary services that address their needs across the medical, psychosocial and legal sectors. They often provide services such as medical care, legal advice, counselling, and safe housing, and they also frequently provide referrals to any services for survivors offered outside the centre.

The remaining three studies examined other interventions, including: Family and Sexual Violence Units in police stations, Family Support Centres in hospitals, a counselling and referral hotline for survivors of VAW, a service referral pathway, and family mediation [25, 26, 46].

### Study design and quality

Four (36%) of the 11 quantitative studies had an overall quality rating of strong, two (18%) were rated moderate, and the remaining five (45%) had a weak quality rating (see Tables 3 and 4). Four (36%) of the studies were a randomised controlled trial (RCT) design or cluster-randomised controlled trial and two (18%) employed a controlled clinical trial design. Four (36%) studies employed a cohort study design 1 or 2 groups. Finally, one study (9%) used a cross-sectional study design.

The methodological quality appraisal of the 11 qualitative studies did not result in an overall score, but instead an assessment of strengths and weaknesses for each study, which are outlined in Table 2. Many of these studies (*n* = 6, 55%) were mixed-methods evaluations that focused on collecting quantitative process data and qualitative outcome data [24, 25, 27–29, 44]. The most common methods used for these evaluations were: reviews of existing reports, policies and other documents; analysis of existing quantitative data; interviews or focus groups with clients, staff and other stakeholders; and site visits or observation. The remaining 5 qualitative studies consisted of data collected only from interviews or focus groups with clients and other stakeholders [26, 42, 43, 45, 46].

### Intervention impact: Alcohol use management (*n* = 3)

Some evidence was found for the effectiveness of psychotherapeutic and counselling interventions when they were designed to reduce alcohol use, both among perpetrators and survivors of VAW. First, a randomised controlled trial conducted in India found that cognitive behavioural therapy (CBT) for men who were being treated for alcohol dependency syndrome (ADS) was effective at reducing violence against their wives in India [39]. Whilst the intervention did not have a significant impact on alcohol dependence in the men (compared to control group), wives reported a significant decrease in spousal violence and symptoms of depression, anxiety and stress when compared to the control group. This result was significant at both 1 and 3 months post-intervention and this study had a moderate quality assessment rating, due to a low participation rate among eligible patients.

A second randomised controlled trial examined the effectiveness of the WHO’s Brief Intervention for Hazardous and Harmful Drinking for female sex workers in Kenya who reported moderate drinking [40]. The study found that the intervention group reported significant decreases in physical violence from both paying and non-paying sexual partners in the last 30 days, 6 months post-intervention, compared to the control group. This study was assessed to be of high quality.

Finally, the last study that examined a counselling intervention for alcohol was a cluster-randomised controlled trial conducted in slums in Mumbai, India [34]. Individual and group counselling sessions focused on marital communication and problem solving were delivered to wives who reported IPV or heavy drinking by their husbands. Whilst there was a decrease in reported IPV in both groups, no statistically significant difference was detected between the intervention and control groups. This study was assessed to be of weak overall quality.

### Intervention impact: Psychotherapy (*n* = 4)

Some evidence was also found for psychotherapeutic interventions when they were used as treatment for survivors of non-partner sexual violence. All four studies found were conducted in the Democratic Republic of Congo (DRC). The well-established psychotherapeutic treatments described above (CPT, CBT and EMDR), were modified to increase cultural relevance and to allow delivery within the local constraints.

First, a cohort study by Bass et al. [31] evaluated the effectiveness of group-based, culturally modified cognitive processing therapy (CPT). Group CPT therapy was highly effective in reducing PTSD, depression and anxiety symptomology, with effects maintained at 6-month follow up. Hall et al. [32] evaluated the effect of the same intervention on social relationships, finding that participation in group therapy was positively associated with increased emotional support seeking, as well as group membership and participation outside of the therapy group. However these effects were not maintained at 6 months follow up, and there was no difference observed for other measures of social capital. Similarly, O’Callaghan et al. [38] implemented a randomised controlled trial of fifteen sessions of trauma-focused cognitive behaviour therapy (TF-CBT), delivered to one group of 24 girls over a five-week period. Again, a highly significant reduction of PTSD, depression and anxiety symptomology was observed in the intervention group compared to a waitlisted control group.

Allon [27] evaluated the use of group EMDR therapy, delivered to groups of 6–8 women by a visiting Israeli doctor. PTSD symptomology was highly significantly decreased immediately after two two-hour group EMDR sessions, however symptom improvement was greater amongst women who received individual EMDR therapy. As there was no follow-up evaluation, it was unknown whether these effects were maintained long-term. Further, as this was not a randomised controlled trial, it is possible that improvements in both groups may have been due to spontaneous recovery over time. However, as the intervention took place over a relatively short time period (3 weeks), this appeared unlikely.

### Intervention impact: Counselling or support groups (*n* = 6)

Six studies were identified which evaluated the effectiveness of five interventions that utilised counselling or support groups [33, 35–37, 42, 43]. Interventions were heterogeneous, with one comprising a skills training program and informal support group [33], while the other five utilised individual and/or group trauma counselling.

In contrast to the other counselling interventions, which aimed to reduce psychological trauma, Hogwood et al. [35] studied a group counselling intervention that aimed to improve life satisfaction of sexual assault survivors who had adolescent children born from rape, by improving their relationships with those children. Groups of ten women who had experienced rape during conflict met fortnightly with one university-trained Rwandan counsellor over a six-month period. The counselling sessions involved discussion of emotional pain and trauma-related symptoms, parenting skills, and disclosure of past rape to children. Sessions were also structured to encourage women to share experiences and develop within-group support networks. Self-reported life satisfaction increased universally and significantly over the course of the intervention, but decreased slightly at three-months post intervention. There was also a significant increase in the number of participants reporting a good relationship with their child, and the number of participants reporting large social support networks. This study was limited methodologically, as it was un-blinded and used self-report measures to quantify effectiveness, meaning participant answers may have been positively biased due to the Hawthorne effect.

A cohort study by Hustache et al. [36] examined the long-term effect of post-rape psychological support delivered to women at a Medecins Sans Frontieres (MSF) clinic in Brazzaville, Republic of the Congo. Individual trauma counselling was offered to patients by a trained psychologist as part of initial post-rape care, with the aim of improving coping mechanisms. Participants received a median of two individual counselling sessions (range 1–4), as desired. Counsellors mainly employed active listening techniques, allowing women to share their experiences. Discussions also focused on coping mechanisms and future plans, and considered social and familial consequences of sexual assault. Global functioning, that is, the psychologist’s judgement of the patient’s overall ability to carry out day-to-day activities, was assessed at each counselling session, and at 1–2 years follow up. The number of women experiencing medium-severe functional impairment was significantly decreased from 89% pre-intervention to 28.6% at the final counselling session, with the effect maintained at follow up. However, one to two years post-treatment, 40% of women continued to experience psychological distress, and one-third reported familial detachment, suggesting that further ongoing interventions may be necessary.

A study by Lekskes et al. [30] compared the effect of two three-month interventions on PTSD symptomology. 58 Liberian women received 8 sessions (or more if desired) of individual trauma counselling delivered by an untrained local counsellor, in combination with an unspecified number of group counselling sessions, which included discussion of stress management, conflict resolution and hygiene practises. The alternative intervention consisted of a combined skills-training and support group, where women learnt income generating skills such as tie dying, and discussed gender issues and sexual violence in an unstructured manner. PTSD symptomology of women who received counselling tended to decrease slightly post-intervention, although the study did not report statistical significance. In contrast, PTSD symptomology increased, on average, amongst members of the support group and the wait-list control group, suggesting that counselling did likely have some effect compared to either of these options. This study was found to be methodologically weak owing to a number of factors, including differences in PSTD symptom score amongst intervention and control groups pre-intervention. Women in the counselling group had higher initial PTSD symptom scores compared to the control group and skills training support group, meaning that differences pre and post-intervention may have been due to spontaneous evolution towards a medium symptom score, and thus the effectiveness of these interventions remains unclear.

A study by Deb et al. [37], examined the effects of counselling on sexually abused girls aged 13–18 in Kolkata, India. The study found a statistically significant difference in aggression between sexually-abused girls who found counselling to be beneficial when compared to sexually-abused girls who did not find counselling to be beneficial. However, this study had major methodological limitations, greatly reducing the utility of their findings. Some of the limitations include that the sexually-abused girls in the study were purposively sampled, and the comparison group was composed of non-sexually abused girls who did not receive any counselling, so the two groups were systematically different. Further, the study was cross-sectional, and statistical analyses did not take clustering into account. For all of these reasons, the effect of counselling on sexually-abused girls’ aggression cannot be adequately assessed from this study.

Two additional qualitative studies evaluated the effect of counselling interventions for survivors of war-related (mostly non-partner) violence. In Sierra Leone, local female social workers provided one-on-one psychosocial support to women following the war [43]. Interviews were conducted with 4 social workers and 6 clients and the counselling techniques used were found to be very different from those used in “Western” psychological models: the social workers focused heavily on spirituality and giving advice. Clients cited the social workers’ advice and support as playing an important role in their recovery. No women mentioned foreign professionals as making an impact on recovery, suggesting that local social work practices are valuable, despite being very different from those used in the West. Finally, a study in Afghanistan provided women who were survivors of war-related violence with group counselling focused on psycho-education and new skills (e.g., problem solving skills) [42]. In group evaluation sessions, over half of participants mentioned that their social life had improved after the intervention, including their interactions with family members and their stress levels. Many participants also cited being happier or an improvement to their health.

### Intervention impact: Crisis Centres (*n* = 6)

Five evaluations of Crisis Centres were conducted using the mixed-methods approach outlined previously, consisting mainly of quantitative process data and qualitative outcome data [24, 27–29, 44]. There is some evidence that Crisis Centres assist survivors to access a broad range of necessary services, including medical, legal and police services. However, the main insights from these evaluations are key lessons learned that can be used to inform better future implementation.

A mixed-methods evaluation of the ISANGE One Stop Centre in a public hospital in Kigali, Rwanda found that the quality of medical and forensic services available to survivors was very high and that linkages with police services were strong [24]. However, limitations of the program included that survivors were required to file police reports before they could access services, data collection was limited, legal aid was inconsistently available for survivors, and staff were not routinely trained.

The Dilaasa Crisis Centres, located at two public hospitals in Mumbai, India, were found to effectively reach low-income and marginalised survivors [44]. Many survivors cited obtaining emotional support from the counselling provided at the centres and some reported improved psychological health. Survivors also stated that the centres helped them to register their complaints with the police. The staff found it challenging to convince the medical personnel that domestic violence is an issue they should be concerned about, but this was being addressed through ongoing training.

One-Stop Centres in Kenya and Zambia were categorized by their location and ownership, and those located inside health facilities and owned by hospitals were found to be better set up to achieve a broader range of legal and health outcomes for survivors [27]. Survivors also reported that they felt the centres were meeting their health needs. However, stronger linkages with outside services and better integration with the legal system were identified areas that require improvement. Another evaluation of Coordinated Response Centres in Zambia found that this model provides survivors with comprehensive services [28].

An evaluation in Nepal used mobile reproductive health camps to identify survivors of VAW and to provide them with comprehensive crisis centre services [29]. 86% of survey respondents in the camps said that services were good and they successfully reached marginalised populations. However, challenges included difficulty filing police reports because of politics and fear of family discord, as well as difficulty locating survivors because of the long wait between the end of conflict and the intervention (5 years).

The final evaluation of Crisis Centres used only qualitative methods, interviewing 21 survivors of domestic violence and 15 stakeholders involved in the centres or related projects [45]. This study took place in Nicaragua and found that the three *casas de la mujer* (women’s centres) resulted in survivors stating that they had new perceptions of women’s roles in society, increased emotional support and knowledge about their health and legal rights, decreased violence by their partners, and increased involvement in programs to help other survivors.

### Intervention impact: Others (*n* = 3)

A study by Human Rights Watch evaluated interventions for VAW in Papua New Guinea (PNG), including: Family and Sexual Violence Units in police stations, Family Support Centres in hospitals, a counselling and referral hotline, and a service referral pathway [26]. Through interviews with survivors, staff and other stakeholders, they found that these interventions have been effective at increasing support for survivors and access to services. However, many are relatively new and some of the remaining challenges include: a lack of awareness about the available services, limited capacity of services (e.g., safe houses, courts) and minimal reach in rural areas, weak law enforcement response, and limited economic opportunities for survivors leaving their partners. Finally, a major challenge they identified was that many service providers did not put the client’s interests first, suggesting better training is needed. A second evaluation of the 17 Family and Sexual Violence Units in police stations in PNG was also conducted, using both interview data and minimal routinely-collected quantitative data [25]. The evaluation found that police responses to VAW in PNG have begun with these units, however, persistent challenges include: a culture of male dominance in the police force, further training is needed on how to provide supportive, non-judgemental services for survivors, and improvements to procedures and the timeliness of response are needed.

One study examined the effectiveness of family mediation in the Democratic Republic of Congo (DRC) through in-depth interviews with participants [46]. Family mediation aims to reintegrate survivors of wartime sexual violence back into their families, who have often rejected them. Reintegrated survivors reported better relationships with their families, improved opportunities for their children, and fewer mental health problems following the mediation. However, challenges still existed, especially in cases where the survivor had a child from her rapist, or the survivor’s partner has remarried. Additional services such as economic support (e.g., children’s tuition, livelihood training, etc.) were cited as potential ways to improve reintegration.

## Discussion

The studies reviewed suggest some interventions that might be effective at reducing future VAW among survivors. As highlighted in the results section, interventions targeting alcohol use, both among perpetrators and survivors, may be effective at reducing VAW and psychotherapy might be effective for survivors of non-partner sexual violence. Some evidence also exists for crisis centres increasing survivors’ access to services, however, evaluations show that careful implementation and assessment of impact on future VAW are needed. Overall, the evidence for all interventions providing secondary and tertiary prevention for survivors of VAW is weak and limitations to this evidence prevent definitive conclusions on what works.

Previous research has found that alcohol consumption is associated with an increased risk of all types of interpersonal violence [47–49]. The association between alcohol and VAW is likely to explain why three studies in this review were alcohol-related interventions. Previous studies in high-income countries have also found programs integrating alcohol dependence treatment and IPV interventions effective when compared to programs that only focused on alcohol dependence or substance abuse [49, 50]. Given this background research, and that two of the studies in this review found alcohol management interventions to be effective at reducing VAW [39, 40], this is an area worthy of further study.

Similarly, psychotherapeutic interventions, including CBT and EMDR, have been shown by previous randomised controlled trials and a meta-analysis to be highly effective in treating post-rape PTSD [51]. Four studies in this review studied psychotherapeutic interventions, using culturally modified treatment protocols, and targeted at survivors of non-partner sexual violence [30–32, 38]. All three interventions (two studies evaluated the same intervention) were highly successful in reducing PTSD symptomology among survivors of non-partner sexual violence, despite being modified for group-delivery. Group-based psychotherapies appear promising for use in low and low-middle income countries, due to their potential to reach large numbers of women. Further, these interventions are low-cost, with the only resources required being paper, pencils and a trained facilitator. Bass et al. [31] demonstrated that a pyramidal supervision system was a viable option in providing ongoing support to multiple local facilitators after they had received training, allowing for implementation of a far reaching intervention, while enabling mobility of program supervisors. In addition, Hall et al. [32] found increased group membership and participation among group therapy participants, suggesting that social support might be improved by group delivery as well. One limitation of this research is that all identified studies of psychotherapeutic interventions were from the Democratic Republic of the Congo, thus the generalizability of results to other cultures is uncertain. Further, it is worth emphasizing that these interventions seem to be most appropriate and effective for survivors of non-partner sexual violence, where, in most cases, the violence has ceased and they are coping with past trauma. Survivors of IPV, on the other hand, are often facing ongoing violence, and thus may not experience the same effects from these interventions.

Examination of the six interventions involving counselling or support groups suggests that the experience and training of the counsellor is likely of high importance in improving mental health outcomes of VAW survivors. Neither the support group nor counselling described by Lekskes et al. [33] utilised a trained counsellor or session facilitator. Although these interventions were sustained over three months, the support group was found to be ineffective, while counselling resulted in a small change in PTSD symptomology. In comparison, treatment of patients by a trained psychologist at an MSF clinic resulted in significant improvement in PTSD symptomology, although counselling was delivered in just two sessions [36]. This finding should be interpreted with caution as no studies in this group were found to be methodologically strong. Trauma counselling alone appears unlikely to be adequate in addressing all psychological and social consequences of sexual violence, as indicated by continued reporting of familial detachment and recurring invasive memories by many participants after having received MSF-delivered counselling. More comprehensive care may be achieved by combining trauma counselling with psychosocial counselling, such as that found by Hogwood et al. [35] to be effective in improving familial relationships. It is worth noting that between psychotherapeutic interventions and counselling interventions targeting treatment of PTSD symptomology, psychotherapies appeared to be the more viable option for effective implementation in resource poor settings. This was because effectiveness of the therapy was maintained even with the use of local non-clinically trained facilitators. Finally, Doucet and Denov [43] raise the point that the background of the person delivering the counselling may matter. In their study, social workers were respected women from their communities, which might have played a role in the intervention’s success in Sierra Leone. Future studies should examine to what degree the counsellor’s background and role in the community impacts the intervention’s effectiveness.

The studies focusing on crisis centres all used qualitative outcome measures and determined that these interventions often met the needs of survivors including legal, psychosocial and health assistance, but this was highly dependent on implementation and the resources available [24, 27–29, 44, 45]. Despite promising outcomes from multi-sectorial programs, this review and previous studies in upper-middle and high income countries [15, 52] have found that there is large variation in quality of services provided. This is largely due to variability in training, staff skills, linkages with other services and infrastructure. Whilst multi-sectorial services through crisis centres show promise, they may not be sustainable in all settings and further evaluation to understand their effects on survivors’ outcomes are needed. Future implementations should take the lessons learned from these qualitative studies into account to ensure that the highest quality services are being offered to survivors.

Weak evidence was found for other interventions, including special units in police stations and family mediation. The studies evaluating these interventions relied on qualitative outcome data, which reported some positive outcomes but also many challenges [25, 26, 46]. One study examining VAW units in police stations in India with quantitative outcome data was excluded from this review because it was a secondary source (citing data from another study and providing no information on methods), and the author informed the reviewers that the primary study is an internal document only [53]. However, the reported results suggest that most women (88.8%) were somewhat or greatly satisfied by the services they received, 40% gained confidence to seek additional services, and 21.5% reported reduced violence, suggesting that this intervention could have some value, but requires further study [54].

The focus of this review on secondary and tertiary prevention, while useful for determining what services work for survivors of VAW, may have excluded primary prevention interventions that could be applicable to survivors, as well as the general population. For example, a randomised controlled trial that was excluded from this review found that gender dialogue groups and group savings (targeted at women over 18, not only survivors of VAW) were effective at reducing IPV, relative to a group savings-only control group [55]. However, the effect disappeared in a sub-analysis of women who were married as children, suggesting the intervention may be less effective among more vulnerable groups [56]. Another RCT that was also excluded, found that women receiving an economic empowerment intervention in Pakistan reported less abuse than counselling and control groups, however, results were not statistically significant [57]. Two excluded cohort studies conducted in Nairobi, Kenya found a self-defence intervention was associated with lower incidence of non-partner sexual violence among adolescent girls [58, 59]. Given that there is some evidence for these interventions among broader populations, it would be valuable to study the effectiveness of these interventions for secondary or tertiary prevention among survivors of VAW.

Recent reviews have examined interventions for women experiencing violence in high income countries. Alcohol management programs for male perpetrators have been shown to result in significant decreases in domestic violence recidivism [60, 61], consistent with our findings. Other interventions that have shown impact on violence in high income countries include women-centred counselling and community-based advocacy and support to access services [15]. Our findings support the effectiveness of some psychotherapeutic interventions for improving wellbeing in non-partner sexual violence in low and low-middle income countries. However, we did not identify evidence for the effectiveness of psychotherapeutic counselling, other forms of counselling or support for accessing services (e.g. through crisis centres) in preventing future intimate partner violence. There is therefore a clear need for future evaluations of such programs in low and low-middle income countries to assess post-intervention experience of violence as an outcome.

This review had several limitations. First, the limited search strategy may have resulted in missed relevant articles. This includes a risk of language bias, as the searches were only conducted in English. We also used a restrictive search strategy in this review to identify studies of high relevance and minimise the number of unrelated results (e.g., use of search terms for “secondary prevention” and “tertiary prevention”, instead of only “prevention”), which again could have led to missed relevant studies. However, many of the articles recommended by experts in the field were identified in the process of database searching, suggesting that most relevant articles were captured. An additional limitation is that data extraction was performed by only one reviewer for several studies (JLW), due to time limitations. However, the use of standardized categories for data extraction and assessment of study quality by multiple reviewers (with high inter-rater reliability), suggests that this was unlikely to cause any problems. Whist this review did have several possible limitations, we attempted to minimise their impact and to mitigate their occurrence. The review methods were developed based on previous reviews in the area [62–64] and according to systematic review guidelines [65–67].

## Conclusions

This systematic review highlights the limited evidence that exists for secondary and tertiary prevention interventions for VAW in low and low-middle income countries. Though some interventions show promise, further research in this area is essential. In particular, more high-quality, outcome-focused studies are needed to determine which interventions work, and for which populations. For example, while we have some qualitative evidence that survivors were able to access services through crisis centres, further study is needed to know to what degree these services lead to positive outcomes for these survivors, and whether they are more effective for certain survivors, so promotion and outreach can be better targeted. This systematic review has highlighted other key areas for future research as well, including continued study of the effectiveness of alcohol-related interventions for both perpetrators and survivors, and psychotherapeutic interventions for survivors of non-partner sexual violence. Better understanding of the effectiveness of these interventions in low and low-middle income countries will help to prioritize programs and policies that reduce the violence, trauma and suffering that many survivors of VAW face around the world.

## Additional file

Additional file 1: Search Terms. This Additional file provides the exact search terms used, organized by database. (DOCX 23 kb)

## Abbreviations

ADS: Alcohol dependency syndrome
ASSIA: Applied Social Sciences Index and Abstracts
CBT: Cognitive behavioural therapy
CPT: Cognitive processing therapy
DRC: Democratic Republic of Congo
EDMR: Eye movement desensitisation and reprocessing
EPHPP-QATQS: Effective Public Health Practice Project Quality Assessment Tool for Quantitative Studies
HIV: Human immunodeficiency virus
IBSS: International Bibliography of the Social Sciences
IPV: Intimate partner violence
MSF: Medecins Sans Frontieres
PNG: Papua New Guinea
PTSD: Post-traumatic stress disorder
RCT: Randomised controlled trial
TF-CBT: Trauma-focused cognitive behaviour therapy
VAW: Violence against women
WHO: World Health Organization
