# Supplementary material for: Effectiveness of secondary and tertiary prevention for violence against women in low and low-middle income countries: a systematic review
Source: BMC Public Health. 2017 Jul 4;17:622. doi: 10.1186/s12889-017-4502-6 (PMC5496243; doi:10.1186/s12889-017-4502-6)
Supplement: Additional file 1: — Search Terms. This Additional file provides the exact search terms used, organized by database. (DOCX 23 kb) [file 12889_2017_4502_MOESM1_ESM.docx]

**Additional file 1: Search Terms**

***Intimate Partner Violence searches:***

Search strategy for Cochrane Database:

#1 ALL: Afghanistan or Benin or Burkina Faso or Burundi or Cambodia or Central Africa or Chad or Comoros or Congo or Eritrea or Ethiopia or Gambia or Guinea or Bisau or Haiti or North Korea or Liberia or Madagascar or Malawi or Mali or Mozambique or Nepal or Niger or Rwanda or Sierra Leone or Somalia or South Sudan or Tanzania or Togo or Uganda or Zimbabwe or Armenia or Bangladesh or Bhutan or Bolivia or Cabo Verde or Cameroon or Congo or Cote d'Ivorie or Ivory Coast or Djibouti or Egypt or El Salvador or Georgia or Ghana or Guatemala or Guyana or Honduras or India or Indonesia or Kenya or Kiribati or Kosovo or Kyrgyz Republic or Lao or Lesotho or Mauritania or Micronesia or Moldova or Morocco or Myanmar or Nicaragua or Nigeria or Pakistan or Papua New Guinea or Philippines or Samoa or Sao Tome or Principe or Senegal or Solomon Islands or Sri Lanka or Sudan or Swaziland or Syrian Arab or Tajikistan or Timor Leste or Ukraine or Uzbekistan or Vanuatu or Vietnam or West Bank or Gaza or Yemen or Zambia

#2 Title, abs, key: (wom?n or female* or girl* or wife or partner or spouse) near/5 (violence or intimate partner violence or sexual abuse or abuse or emotional abuse or physical abuse or physical violence or sexual violence or neglect or gender violence or domestic violence or physical partner violence or sexual partner violence)

#3 ALL: (treatment or "secondary prevention" or "tertiary prevention" or intervention)

#4 ALL: (evalua* or effectiv* or efficacy or outcome or what works or outcome or trial*)

1 and 2 and 3 and 4

Search strategy for Medline and PsychINFO:

#1: (Afghanistan or Benin or Burkina Faso or Burundi or Cambodia or Central Africa or Chad or Comoros or Congo or Eritrea or Ethiopia or Gambia or Guinea or Bisau or Haiti or North Korea or Liberia or Madagascar or Malawi or Mali or Mozambique or Nepal or Niger or Rwanda or Sierra Leone or Somalia or South Sudan or Tanzania or Togo or Uganda or Zimbabwe).mp.

#2: (Armenia or Bangladesh or Bhutan or Bolivia or Cabo Verde or Cameroon or Congo or Cote d'Ivorie or Ivory Coast or Djibouti or Egypt or El Salvador or Georgia or Ghana or Guatemala or Guyana or Honduras or India or Indonesia or Kenya or Kiribati or Kosovo or Kyrgyz Republic or Lao or Lesotho or Mauritania or Micronesia or Moldova or Morocco or Myanmar or Nicaragua or Nigeria or Pakistan or Papua New Guinea or Philippines or Samoa or Sao Tome or Principe or Senegal or Solomon Islands or Sri Lanka or Sudan or Swaziland or Syrian Arab or Tajikistan or Timor Leste or Ukraine or Uzbekistan or Vanuatu or Vietnam or West Bank or Gaza or Yemen or Zambia).mp.

#3: ((wom$n or female* or girl* or wife or partner or spouse) adj5 (violence or intimate partner violence or sexual abuse or abuse or emotional abuse or physical abuse or physical violence or sexual violence or neglect or gender violence or domestic violence or physical partner violence or sexual partner violence)).ab.

#4: (treatment or "secondary prevention" or "tertiary prevention" or intervention).mp.

#5: (1 or 2) and 3 and 4

#6: (evalua* or effectiv* or efficacy or outcome or what works or outcome or trial*).mp.

#7: 5 and 6

Search strategy for ASSIA, IBSS and Sociological abstracts:

ab((wom?n OR female* OR girl* OR wife OR partner OR spouse) NEAR/5 (violence OR abuse OR neglect))

AND (treatment OR "secondary prevention" OR "tertiary prevention" OR intervention)

AND (Afghanistan OR Benin OR Burkina Faso OR Burundi OR Cambodia OR Central Africa OR Chad OR Comoros OR Congo OR Eritrea OR Ethiopia OR Gambia OR Guinea OR Bisau OR Haiti OR North Korea OR Liberia OR Madagascar OR Malawi OR Mali OR Mozambique OR Nepal OR Niger OR Rwanda OR Sierra Leone OR Somalia OR South Sudan OR Tanzania OR Togo OR Uganda OR Zimbabwe OR Armenia OR Bangladesh OR Bhutan OR Bolivia OR Cabo Verde OR Cameroon OR Congo OR Cote d'Ivorie OR Ivory Coast OR Djibouti OR Egypt OR El Salvador OR Georgia OR Ghana OR Guatemala OR Guyana OR Honduras OR India OR Indonesia OR Kenya OR Kiribati OR Kosovo OR Kyrgyz Republic OR Lao OR Lesotho OR Mauritania OR Micronesia OR Moldova OR Morocco OR Myanmar OR Nicaragua OR Nigeria OR Pakistan OR Papua New Guinea OR Philippines OR Samoa OR Sao Tome OR Principe OR Senegal OR Solomon Islands OR Sri Lanka OR Sudan OR Swaziland OR Syrian Arab OR Tajikistan OR Timor Leste OR Ukraine OR Uzbekistan OR Vanuatu OR Vietnam OR West Bank OR Gaza OR Yemen OR Zambia)

AND (evalua* OR effectiv* OR efficacy OR outcome OR what works OR trial*)

Search strategy for Web of Science^[[1]](#footnote-1)^:

#1 TS: (Afghanistan or Benin or Burkina Faso or Burundi or Cambodia or Central Africa or Chad or Comoros or Congo or Eritrea or Ethiopia or Gambia or Guinea or Bisau or Haiti or North Korea or Liberia or Madagascar or Malawi or Mali or Mozambique or Nepal or Niger or Rwanda or Sierra Leone or Somalia or South Sudan or Tanzania or Togo or Uganda or Zimbabwe or Armenia or Bangladesh or Bhutan or Bolivia or Cabo Verde or Cameroon or Congo or Cote d'Ivorie or Ivory Coast or Djibouti or Egypt or El Salvador or Georgia or Ghana or Guatemala or Guyana or Honduras or India or Indonesia or Kenya or Kiribati or Kosovo or Kyrgyz Republic or Lao or Lesotho or Mauritania or Micronesia or Moldova or Morocco or Myanmar or Nicaragua or Nigeria or Pakistan or Papua New Guinea or Philippines or Samoa or Sao Tome or Principe or Senegal or Solomon Islands or Sri Lanka or Sudan or Swaziland or Syrian Arab or Tajikistan or Timor Leste or Ukraine or Uzbekistan or Vanuatu or Vietnam or West Bank or Gaza or Yemen or Zambia)

#2 TI: ((wom?n or female* or girl* or wife or partner or spouse) NEAR/5 (violence or abuse or neglect))

#3 TS: (treatment or "secondary prevention" or "tertiary prevention" or intervention)

#4 TS: (evalua* or effectiv* or efficacy or outcome or what works or outcome or trial*)

#1 AND #2 AND #3 AND #4

Search strategy for Global Index Medicus^[[2]](#footnote-2)^

(tw:((domestic violence or intimate partner violence or violence against women or gender based violence or gender violence or physical partner violence or sexual partner violence)))

AND (tw:((treatment or "secondary prevention" or "tertiary prevention" or intervention or interventions or treatments)))

Search strategy for CINAHL:

(women OR female OR girl OR wife OR partner OR spouse )

AND ( treatment OR "secondary prevention" OR "tertiary prevention" OR intervention )

AND ( evaluation OR effective OR efficacy OR outcome OR what works OR outcome OR trial )

AND ( Afghanistan OR Benin OR Burkina Faso OR Burundi OR Cambodia OR Central Africa OR Chad OR Comoros OR Congo OR Eritrea OR Ethiopia OR Gambia OR Guinea OR Bisau OR Haiti OR North Korea OR Liberia OR Madagascar OR Malawi OR Mali OR Mozambique OR Nepal OR Niger OR Rwanda OR Sierra Leone OR Somalia OR South Sudan OR Tanzania OR Togo OR Uganda OR Zimbabwe OR Armenia OR Bangladesh OR Bhutan OR Bolivia OR Cabo Verde OR Cameroon OR Congo OR Cote d'Ivorie OR Ivory Coast OR Djibouti OR Egypt OR El Salvador OR Georgia OR Ghana OR Guatemala OR Guyana OR Honduras OR India OR Indonesia OR Kenya OR Kiribati OR Kosovo OR Kyrgyz Republic OR Lao OR Lesotho OR Mauritania OR Micronesia OR Moldova OR Morocco OR Myanmar OR Nicaragua OR Nigeria OR Pakistan OR Papua New Guinea OR Philippines OR Samoa OR Sao Tome OR Principe OR Senegal OR Solomon Islands OR Sri Lanka OR Sudan OR Swaziland OR Syrian Arab OR Tajikistan OR Timor Leste OR Ukraine OR Uzbekistan OR Vanuatu OR Vietnam OR West Bank OR Gaza OR Yemen OR Zambia ) AND ( violence OR intimate partner violence OR sexual abuse OR abuse OR emotional abuse OR physical abuse OR physical violence OR sexual violence OR neglect OR gender violence OR domestic violence OR physical partner violence OR sexual partner violence )

Search strategy for Embase:

(('women' OR 'female' OR 'girl' OR 'wife' OR 'partner' OR 'spouse') NEAR/5 ('violence' OR 'abuse' OR 'neglect')):ab,ti AND ('treatment' OR 'secondary prevention'/exp OR 'secondary prevention' OR 'tertiary prevention'/exp OR 'tertiary prevention' OR 'intervention')

AND ('afghanistan'/exp OR 'afghanistan' OR 'benin'/exp OR 'benin'OR 'burkina faso'/exp OR 'burkina faso' OR 'burundi'/exp OR 'burundi' OR 'cambodia'/exp OR 'cambodia' OR 'central africa'/exp OR 'central africa' OR 'chad'/exp OR'chad' OR 'comoros'/exp OR 'comoros' OR 'congo'/exp OR 'congo' OR 'eritrea'/exp OR 'eritrea' OR 'ethiopia'/exp OR 'ethiopia' OR 'gambia'/exp OR 'gambia' OR'guinea'/exp OR 'guinea' OR 'haiti'/exp OR 'haiti' OR 'north korea'/exp OR 'north korea' OR 'liberia'/exp OR 'liberia' OR 'madagascar'/exp OR 'madagascar' OR'malawi'/exp OR 'malawi' OR 'mali'/exp OR 'mali' OR 'mozambique'/exp OR 'mozambique' OR 'nepal'/exp OR 'nepal' OR 'niger'/exp OR 'niger' OR 'rwanda'/exp OR'rwanda' OR 'sierra leone'/exp OR 'sierra leone' OR 'somalia'/exp OR 'somalia' OR 'south sudan'/exp OR 'south sudan' OR 'tanzania'/exp OR 'tanzania' OR 'togo'/exp OR 'togo' OR 'uganda'/exp OR 'uganda' OR 'zimbabwe'/exp OR 'zimbabwe' OR 'armenia'/exp OR 'armenia' OR 'bangladesh'/exp OR 'bangladesh' OR 'bhutan'/exp OR'bhutan' OR 'bolivia'/exp OR 'bolivia' OR 'cameroon'/exp OR 'cameroon' OR 'ivory coast'/exp OR 'ivory coast' OR 'djibouti'/exp OR 'djibouti' OR 'egypt'/exp OR 'egypt'OR 'el salvador'/exp OR 'el salvador' OR 'georgia (republic)'/exp OR 'georgia (republic)' OR 'ghana'/exp OR 'ghana' OR 'guatemala'/exp OR 'guatemala' OR'guyana'/exp OR 'guyana' OR 'honduras'/exp OR 'honduras' OR 'india'/exp OR 'india' OR 'indonesia'/exp OR 'indonesia' OR 'kenya'/exp OR 'kenya' OR 'kiribati'/exp OR'kiribati' OR 'kosovo'/exp OR 'kosovo' OR 'kyrgyzstan'/exp OR 'kyrgyzstan' OR 'laos'/exp OR 'laos' OR 'lesotho'/exp OR 'lesotho' OR 'mauritania'/exp OR 'mauritania'OR 'micronesia'/exp OR 'micronesia' OR 'moldova'/exp OR 'moldova' OR 'morocco'/exp OR 'morocco' OR 'myanmar'/exp OR 'myanmar' OR 'nicaragua'/exp OR'nicaragua' OR 'nigeria'/exp OR 'nigeria' OR 'pakistan'/exp OR 'pakistan' OR 'papua new guinea'/exp OR 'papua new guinea' OR 'philippines'/exp OR 'philippines' OR'samoa'/exp OR 'samoa' OR 'sao tome and principe'/exp OR 'sao tome and principe' OR 'senegal'/exp OR 'senegal' OR 'solomon islands'/exp OR 'solomon islands' OR'sri lanka'/exp OR 'sri lanka' OR 'sudan'/exp OR 'sudan' OR 'swaziland'/exp OR 'swaziland' OR 'syrian arab republic'/exp OR 'syrian arab republic' OR 'tajikistan'/exp OR 'tajikistan' OR 'timor-leste'/exp OR 'timor-leste' OR 'ukraine'/exp OR 'ukraine' OR 'uzbekistan'/exp OR 'uzbekistan' OR 'vanuatu'/exp OR 'vanuatu' OR 'vietnam'/exp OR 'vietnam' OR 'gaza strip palestine' OR 'yemen'/exp OR 'yemen' OR 'zambia'/exp OR 'zambia')

AND ('evaluation'/exp OR 'evaluation' OR 'effective' OR 'efficacy' OR'outcome' OR 'trial')

***Non-Partner Sexual Violence searches:***

The search strategy was shortened in some databases due to problems with the web interface. Shortening of the search strategy resulted in a greater number of articles retrieved from databases, which were then reviewed by abstract, so did not detract from the comprehensiveness of the search.

Search strategy for Medline and PsychINFO:

#1: (sexual abuse or sexual assault or sexual violence or sexual coercion or rape or sexual harassment).tw

#2: (Intervention or treatment or response or best practice or secondary prevention

or tertiary prevention).mp

#3: (evaluat* or what works or efficacy or effective or effectiveness or impact or outcome or measure).mp

#4: (women or girl* or child* or female).mp

#5: (Afghanistan or Benin or "Burkina Faso" or Burundi or Cambodia or "Central African Republic" or Chad or Comoros or "Democratic republic of the Congo" or Congo or Eritrea or Ethiopia or Gambia or Guinea or Guinea-Bisau or Haiti or Liberia or Madagascar or Malawi or Mali or Mozambique or Nepal or Niger or Rwanda or "Sierra Leone" or Somalia or "South Sudan" or Tanzania or Togo or Uganda or Zimbabwe or Armenia or Bangladesh or Bhutan or Bolivia or "Cabo Verde" or Cameroon or Congo or "Cote d Ivoire" or Djibouti or Egypt or "El Salvador" or Georgia or Ghana or Guatemala or Guyana or Honduras or India or Indonesia or Kenya or Kiribati or Kosovo or "Kyrgyz Republic" or Laos or Lesotho or Mauritania or Micronesia or Moldova or Morocco or Myanmar or Nicaragua or Nigeria or Pakistan or "Papua New Guinea" or Philippines or Samoa or "Sao Tome" or Principe or Senegal or "Solomon islands" or "Sri Lanka" or Sudan or Swaziland or "Syrian Arab Republic" or Tajikistan or Timor-Leste or Ukraine or Uzbekistan or Vanuatu or Vietnam or "West Bank" or Gaza or Yemen or Zambia).mp

#1 AND #2 AND #3 AND #4 AND #5

Search strategy for Cochrane, CINAHL and Embase:

(sexual abuse OR sexual assault OR sexual violence OR sexual coercion OR rape OR sexual harassment)

AND (Intervention OR treatment OR response OR best practice OR secondary prevention

OR tertiary prevention)

AND (evaluation OR evaluate OR what works OR efficacy OR effective OR effectiveness OR impact OR outcome OR measure)

AND (women OR girls OR girl OR child OR children OR female)

AND (Afghanistan OR Benin OR “Burkina Faso” OR Burundi OR Cambodia OR “Central African Republic” OR Chad OR Comoros OR “Democratic republic of the Congo” OR Congo OR Eritrea OR Ethiopia OR Gambia OR Guinea OR Guinea-Bisau OR Haiti OR Liberia OR Madagascar OR Malawi OR Mali OR Mozambique OR Nepal OR Niger OR Rwanda OR “Sierra Leone” OR Somalia OR “South Sudan” OR Tanzania OR Togo OR Uganda OR Zimbabwe OR Armenia OR Bangladesh OR Bhutan OR Bolivia OR “Cabo Verde” OR Cameroon OR Congo OR “Cote d Ivoire” OR Djibouti OR Egypt OR "El Salvador" OR Georgia OR Ghana OR Guatemala OR Guyana OR Honduras OR India OR Indonesia OR Kenya OR Kiribati OR Kosovo OR "Kyrgyz Republic" OR Laos OR Lesotho OR Mauritania OR Micronesia OR Moldova OR Morocco OR Myanmar OR Nicaragua OR Nigeria OR Pakistan OR "Papua New Guinea" OR Philippines OR Samoa OR "Sao Tome" OR Principe OR Senegal OR "Solomon islands" OR "Sri Lanka" OR Sudan OR Swaziland OR "Syrian Arab Republic" OR Tajikistan OR Timor-Leste OR Ukraine OR Uzbekistan OR Vanuatu OR Vietnam OR "West Bank" OR Gaza OR Yemen OR Zambia)

Search strategy for Sociological Abstracts and Applied Social Sciences Index:

TI(sexual abuse OR sexual assault OR sexual violence OR sexual coercion OR rape OR sexual harassment)

AND AB(Intervention OR treatment OR response OR best practice OR secondary prevention

OR tertiary prevention)

AND AB(evaluat* OR what works OR efficacy OR effective OR effectiveness OR impact OR outcome OR measure)

AND AB(women OR girl* OR child* OR female)

AND AB(Afghanistan OR Benin OR “Burkina Faso” OR Burundi OR Cambodia OR “Central African Republic” OR Chad OR Comoros OR “Democratic republic of the Congo” OR Congo OR Eritrea OR Ethiopia OR Gambia OR Guinea OR Guinea-Bisau OR Haiti OR Liberia OR Madagascar OR Malawi OR Mali OR Mozambique OR Nepal OR Niger OR Rwanda OR “Sierra Leone” OR Somalia OR “South Sudan” OR Tanzania OR Togo OR Uganda OR Zimbabwe OR Armenia OR Bangladesh OR Bhutan OR Bolivia OR “Cabo Verde” OR Cameroon OR Congo OR “Cote d Ivoire” OR Djibouti OR Egypt OR "El Salvador" OR Georgia OR Ghana OR Guatemala OR Guyana OR Honduras OR India OR Indonesia OR Kenya OR Kiribati OR Kosovo OR "Kyrgyz Republic" OR Laos OR Lesotho OR Mauritania OR Micronesia OR Moldova OR Morocco OR Myanmar OR Nicaragua OR Nigeria OR Pakistan OR "Papua New Guinea" OR Philippines OR Samoa OR "Sao Tome" OR Principe OR Senegal OR "Solomon islands" OR "Sri Lanka" OR Sudan OR Swaziland OR "Syrian Arab Republic" OR Tajikistan OR Timor-Leste OR Ukraine OR Uzbekistan OR Vanuatu OR Vietnam OR "West Bank" OR Gaza OR Yemen OR Zambia)

Search strategy for WHO Global Health Library:

#1: (sexual abuse or sexual assault or sexual violence or sexual coercion or rape or sexual harassment).tw

#2: (Intervention or treatment or response or best practice or secondary prevention

or tertiary prevention).tw

#3: (evaluat* or what works or efficacy or effective or effectiveness or impact or outcome or measure).tw

#4: (Afghanistan or Benin or "Burkina Faso" or Burundi or Cambodia or "Central African Republic" or Chad or Comoros or "Democratic republic of the Congo" or Congo or Eritrea or Ethiopia or Gambia or Guinea or Guinea-Bisau or Haiti or Liberia or Madagascar or Malawi or Mali or Mozambique or Nepal or Niger or Rwanda or "Sierra Leone" or Somalia or "South Sudan" or Tanzania or Togo or Uganda or Zimbabwe or Armenia or Bangladesh or Bhutan or Bolivia or "Cabo Verde" or Cameroon or Congo or "Cote d Ivoire" or Djibouti or Egypt or "El Salvador" or Georgia or Ghana or Guatemala or Guyana or Honduras or India or Indonesia or Kenya or Kiribati or Kosovo or "Kyrgyz Republic" or Laos or Lesotho or Mauritania or Micronesia or Moldova or Morocco or Myanmar or Nicaragua or Nigeria or Pakistan or "Papua New Guinea" or Philippines or Samoa or "Sao Tome" or Principe or Senegal or "Solomon islands" or "Sri Lanka" or Sudan or Swaziland or "Syrian Arab Republic" or Tajikistan or Timor-Leste or Ukraine or Uzbekistan or Vanuatu or Vietnam or "West Bank" or Gaza or Yemen or Zambia).tw

#1 AND #2 AND #3 AND #4

Search strategy for Web of Science:

TI=(sexual abuse OR sexual assault OR sexual violence OR sexual coercion OR rape OR sexual harassment)

AND TS=(Intervention OR treatment OR response OR best practice OR secondary prevention

OR tertiary prevention)

AND TS=(women OR girl* OR child* OR female)

AND TS=(Afghanistan OR Benin OR “Burkina Faso” OR Burundi OR Cambodia OR “Central African Republic” OR Chad OR Comoros OR “Democratic republic of the Congo” OR Congo OR Eritrea OR Ethiopia OR Gambia OR Guinea OR Guinea-Bisau OR Haiti OR Liberia OR Madagascar OR Malawi OR Mali OR Mozambique OR Nepal OR Niger OR Rwanda OR “Sierra Leone” OR Somalia OR “South Sudan” OR Tanzania OR Togo OR Uganda OR Zimbabwe OR Armenia OR Bangladesh OR Bhutan OR Bolivia OR “Cabo Verde” OR Cameroon OR Congo OR “Cote d Ivoire” OR Djibouti OR Egypt OR "El Salvador" OR Georgia OR Ghana OR Guatemala OR Guyana OR Honduras OR India OR Indonesia OR Kenya OR Kiribati OR Kosovo OR "Kyrgyz Republic" OR Laos OR Lesotho OR Mauritania OR Micronesia OR Moldova OR Morocco OR Myanmar OR Nicaragua OR Nigeria OR Pakistan OR "Papua New Guinea" OR Philippines OR Samoa OR "Sao Tome" OR Principe OR Senegal OR "Solomon islands" OR "Sri Lanka" OR Sudan OR Swaziland OR "Syrian Arab Republic" OR Tajikistan OR Timor-Leste OR Ukraine OR Uzbekistan OR Vanuatu OR Vietnam OR "West Bank" OR Gaza OR Yemen OR Zambia)

Search strategy for Greylit:

(Sexual violence OR sexual abuse OR sexual assault) AND (response OR intervention OR treatment)

Search strategy for Opengrey:

(sexual abuse OR sexual assault OR sexual violence OR sexual coercion OR rape OR sexual harassment)

AND (Intervention OR treatment OR response OR best practice OR secondary prevention

OR tertiary prevention)

AND (evaluat* OR what works OR efficacy OR effective OR effectiveness OR impact OR outcome OR measure)

1. Where TS denotes a “topic” search of the whole article and TI denotes a title search. [↑](#footnote-ref-1)
2. Where TW denotes a title, abstract and subject search [↑](#footnote-ref-2)
